# *Nao-Xue-Shu* Oral Liquid Protects and Improves Secondary Brain Insults of Hypertensive Cerebral Hemorrhage

**DOI:** 10.1155/2016/9121843

**Published:** 2016-03-27

**Authors:** Hongning Jiang, Ying Qin, Te Liu, Liang Zhang, Mingzhe Wang, Baofeng Qin, Wenfei Jiang, Weilong Liao, Weidong Pan

**Affiliations:** ^1^Department of Radiology, Shuguang Hospital, Shanghai Traditional Chinese Medicine, Shanghai 201203, China; ^2^Department of Respiratory Medicine, Pudong Hospital of Traditional Chinese Medicine, Shanghai 201209, China; ^3^Shanghai Geriatric Institute of Chinese Medicine, Longhua Hospital, Shanghai University of Traditional Chinese Medicine, Shanghai 200031, China; ^4^Department of Neurology, Shanghai Seventh Hospital, Shanghai University of Traditional Chinese Medicine, Shanghai 200137, China; ^5^Department of Neurology, Shuguang Hospital, Shanghai Traditional Chinese Medicine, Shanghai 201203, China

## Abstract

*Aim.* To determine one traditional Chinese medicine (TCM)* Nao-Xue-Shu* oral liquid which protects and improves secondary brain insults (SBI) in hypertensive cerebral hemorrhage (HCH).* Methods.* 158 patients with HCH were divided into routine clinical medicine plus* Nao-Xue-Shu* oral liquid (*n* = 78) as treatment group, and routine clinical medicine (*n* = 80) only served as the control group. The incidence of SBI and the classification of a favorable prognosis and a bad prognosis using the Glasgow outcome scale (GOS) were assessed to evaluate the clinical effects. The changes of IL-6 and TNF-*α* levels were determined to study the mechanism of the effects for the TCM.* Results.* The incidence of SBI at the end of week 2 was 8.97% in the treatment group and 23.75% in the control group, and the difference was significant (*P* < 0.001). The incidence of a favorable prognosis was 48.72% in the treatment group and 32.72% in the control group, and the difference was significant (*P* < 0.01) at the end of week 2. These findings indicate clear differences for IL-6 and TNF-*α* at the end of week 1 and week 2 compared with before treatment for the treatment group and a marked difference at the end of week 2 between the two groups. It also shows a significant difference between the end of week 2 and before treatment for IL-6 and TNF-*α* for the control group, although the difference was much smaller than the treatment group.* Conclusion. Nao-Xue-Shu* oral liquid could protect against the occurrence of SBI and improve HCH and SBI patients. It may also decrease the damage and the mass effects of the hematoma by reducing IL-6 and TNF-*α* to obtain the effects, and thus it is a potentially suitable drug for HCH and SBI.

## 1. Introduction

Secondary brain insults (SBI) predominantly due to hypotension are frequent among patients with fatal traumatic brain injury. SBI following primary impact (e.g., secondary hemorrhage insults) are important causes of damage to the brain [1, 2]. If the SBI is caused by hypertensive cerebral hemorrhage (HCH), it will be defined as hypertensive cerebral hemorrhage SBI (HCH-SBI). In the past two decades, including animal experiments and clinical trials, a great number of studies have been carried out on HCH-SBI. It has been shown that hypotension or hypoxia [3, 4], intraoperative hypotension [5], and hyperglycemia in the intensive care unit of HCH-SBI are consistently associated with poor outcomes in HCH patients. Hypotension is one of the most significant symptoms of SBI after head injuries [6]. The combination of hypotension and head injury is associated with increased mortality and morbidity in comparison with hypertensive cerebral hemorrhage alone [7]. Despite animal studies indicating encouraging results, however, human trials assessing the use of pharmacological agents after SBI have all failed to show efficacy. Proinflammatory factors such as serum interleukin 6 (IL-6) and tumor necrosis factor *α* (TNF-*α*) may indicate potential mechanism of the brain injury [8] and may investigate pathogenesis of HCH-SBI.* Nao-Xue-Shu* oral liquid [9], a traditional Chinese medicine (TCM), is often used to treat HCH in China and decrease the incidence of SBI. Therefore, the current study is directed towards providing an optimal physiological environment in order to minimize SBI and maximize the body's own regenerative processes.

## 2. Subjects and Methods

### 2.1. Subjects

A total of 158 patients with HCH from our two hospitals (93 cases from Shuguang Hospital and 65 from Shanghai Seventh Hospital) were collected to participate in the study. All of the patients had been diagnosed with hypertension before suffering from a cerebral hemorrhage and 147 cases had a higher blood pressure level than normal when the cerebral hemorrhage occurred. The remaining 11 cases were excluded due to a second cerebral hemorrhage, such as an arterial aneurysm, moyamoya disease, cerebral arteriovenous malformation, cerebral venous sinus thrombosis, and CADASIL (cerebral autosomal dominant arteriopathy with subcortical infarcts and leukoencephalopathy) and were classified as having HCH. The cerebral hemorrhage was the first in 151 cases and the second in 7 cases. Intracerebral hemorrhage of the patients was confirmed by computed tomography (CT). The size of an intracerebral hematoma was calculated using the *ABC*/2 formula [2], as follows: the volume of an ellipsoid is 4/3*π*(*A*/2)(*B*/2)(*C*/2), where *A*, *B*, and *C* are the three diameters. If *π* is estimated to be 3, then the volume of an ellipsoid becomes *ABC*/2. Patients were divided into the treatment group (treatment plus* Nao-Xue-Shu* oral liquid, *n* = 78) and control group (treatment without* Nao-Xue-Shu* oral liquid, *n* = 80) in a single-blind fashion. No significant differences in gender, age, number of cases, volume of hematoma, duration, or types of diseases between the two groups were found, and the 2 groups were comparable (Table 1). Inclusion criteria for the patients with SBI were (1) high temperature ≥ 39 degrees for more than 4 hours; (2) blood pressure ≤ 90/60 mmHg for more than 2 hours; (3) oxygen partial pressure (PaO_2_) ≤ 60 mmHg; (4) fasting blood glucose ≥ 9 mmol/L; and (5) electrolyte disorder and acid-base imbalance. If the patients suffered symptoms or signs or had more than 3 changes in laboratory values such as blood pressure and PaO_2_, the patient was defined as suffering from SBI.

### 2.2. Treatment Methods

The control group underwent routine clinical treatments and measurements according to the guidelines of Western medicine [10], including monitoring electrocardiograph (ECG) and blood pressure fluctuations. To control blood pressure and intracranial pressure,* mannitol* and/or* furosemide* and* citicoline* were administered by intravenous infusion according to the patient's situation and the blood pressure was maintained at less than 180/90 mmHg. The patients in the treatment group were treated using the same routine treatments as the control group and were also given 10 mL of* Nao-Xue-Shu* oral liquid [9, 11], three times per day (Shandong* Wohua* Pharmaceutical Polytron Technologies Inc.), which consists of* Astragalus root, Hirudo, Acorus gramineus, radix achyranthis bidentatae, tree peony bark, Rheum officinale*, and* Ligusticum wallichii* (batch number: 5040507 and 5040711). Patients who could not ingest the liquid orally were given it by nasal feeding. The patients in the treatment group took* Nao-Xue-Shu* oral liquid for 2 weeks. The clinical and laboratory parameters were measured before treatment (baseline) and at the end of week 1 and week 2 to evaluate the effects of treatment in the two groups.

### 2.3. Observation Items and Assessments

The blood pressure, respiration, heartbeat, temperature, and arterial oxygen saturation (SpO2) of most patients were monitored with an electrocardiograph. Blood gas analysis and blood sugar were tested once per day. The Glasgow Outcome Scale (GOS) and the incidence of SBI were the main outcome measures of the study. The serum interleukin 6 (IL-6) and tumor necrosis factor *α* (TNF-*α*) levels were determined 3 times (before treatment, at the first weekend, and at the second weekend) as the secondary outcomes. Liver and kidney function were also tested to monitor the side effects of the* Nao-Xue-Shu* oral liquid.

The Glasgow Outcome Score is used to assess patients with brain damage and enables the objective assessment of their recovery into 5 categories. The score is used to predict the long-term course of rehabilitation to return to work and everyday life [12]. It has five degrees: the first degree (I) death: severe injury or death without recovery of consciousness; the second degree (II) persistent vegetative state: severe damage with prolonged state of unresponsiveness and a lack of higher mental functions; the third degree (III) severe disability: severe injury with permanent need for help with daily living; the fourth degree (IV) moderate disability: no need for assistance in everyday life and employment possible but may require special equipment; and the fifth degree (V) low disability: light damage with minor neurological and psychological deficits. We defined the prognosis into a poor prognosis (I–III) group and a favorable prognosis (IV-V) group.

### 2.4. Statistics

SPSS 18.0 software package was used for statistical analysis. Data are presented as the mean and standard deviation (−*x* + *s*) or percentage (%). Chi-square test was used to test the differences of sex and area of the cerebral hemorrhage; *t*-test was conducted to check the differences of age, blood pressure, and volumes of the hematoma of the baseline of the two groups. Repeated-measure ANOVA was conducted to test the differences among changes in outcomes at baseline and at the end of week 1 and week 2 for both groups followed by post hoc Fisher test whenever necessary. Differences of the IL-6 and TNF-*α* levels and the volume of the hematoma were obtained by the questionnaire and were assessed using the Friedman test with subsequent post hoc verification using Wilcoxon test. *P* < 0.05 was considered to indicate a statistically significant difference.

## 3. Results

No significant differences in age, sex, baseline of IL-6, or baseline of TNF-*α* were found between the two groups, and there were no significant differences in liver laboratory tests and kidney functions among baseline, week 1, and week 2 between the treatment and control groups (Tables 1 and 2). At the end of week 2, there were 12 deaths: 4 patients in the treatment group (who died on days 5, 7, 10, and 11) and 8 patients in the control group (2 died on day 2, 2 on day 5, 3 on day 7, and one on day 10).

After two weeks of treatment, 7 patients (8.97%) in the treatment group and 19 (23.75%) in the control group had SBI, and the difference between the two groups was significant (*P* < 0.001, Ridit analyses). During the weekend of week 2, the GOS outcome was a favorable prognosis in 38 cases (48.72%) in the treatment group but in only 25 cases (31.25%) in the control group, and the difference was significant (Ridit analysis, *P* < 0.01). At the end of the study, no significant differences were found such as temperature, blood pressure, oxygen partial pressure, fasting blood glucose, and electrolyte disorder and acid-base imbalance compared between the two groups.

Compared with baseline, IL-6 and TNF-*α* had decreased at the end of week 1 and week 2 in the treatment group (*P* < 0.05 for both laboratory factors of week 1 and *P* < 0.001 for both laboratory factors at the end of week 2), while the factors in the control group only indicated a significantly decreased outcome at the end of week 2 compared with its baseline (*P* < 0.05 for IL-6 and *P* < 0.01 for TNF-*α*), and the decreased level at the end of week 2 was much smaller than that in the treatment group (*P* < 0.001 for both factors, Table 2). The same differences were also found when comparing the two groups by considering the volume of hematoma of the cerebral hemorrhage. The volume of the hematoma had decreased at the end of week 1 and week 2 in the treatment group, while the volume in the control group only indicated a significantly decreased outcome at the end of week 2 compared with its baseline, and the decreased level at the end of week 2 was much smaller than that in the treatment group (Table 2).

## 4. Discussion

Our results indicated that, compared with the control group, the treatment group (*Nao-Xue-Shu* oral liquid) had a lower incidence of SBI at the end of week 2. Furthermore, the treatment group exhibited clinical improvements in the patients with HCH-SBI, indicating that* Nao-Xue-Shu* oral liquid can be used as an additional treatment for HCH and HCH-SBI.

HCH is a common stroke of cerebral hemorrhage in China, has a higher disability rate and higher mortality rate, and is extremely difficult to prevent and treat by modern medicine. Integrative treatment may have additional effects in treating serious and difficult diseases [13–15] such as the occurrence of SBI of HCH. This study demonstrated that* Nao-Xue-Shu* oral liquid could significantly decrease the toxicity of hematoma in the brain by removing IL-6 and TNF-*α* (Table 2), preventing the occurrence of SBI and improving the recovery of neuronal function, and, finally, decreasing the disability and mortality rates of the patients.

SBI was first defined by Miller et al. who studied the insults due to traumatic brain injury [1]. They suggested that the higher disability rate and higher mortality rate of brain injury were not only caused by the traumatic brain injury directly, but also derived from SBI, the mass effect of the hematoma, and peripheral toxicity from the hematoma. Matsushita et al. hypothesized that cell death after intracerebral hemorrhage may be mediated in part by apoptotic mechanisms. They provided initial evidence that apoptotic mechanisms may mediate some of the injury in brain after intracerebral hemorrhage [16]. Xu et al. observed SBI induced by traumatic brain injury, including excitotoxicity, oxidative stress, inflammatory response, and neuronal degeneration, and indicated that mouse brain with traumatic brain injury can be protected by inhibiting the inflammatory response and that inhibiting inflammatory-induced autophagy may play a pivotal role in its neuroprotection [8]. Zhang et al. reported the levels of serum IL-6 and TNF-*α* were increased significantly during the early stage of HCH [17]. Suzuki et al. investigated the pathogenesis of hypertensive cerebrovascular lesions by immunohistochemistry and scanning electron microscopy. The brains of rats with experimentally induced hypertension exhibited severe edema and intracerebral hemorrhage. They found that IL-6, IL-8, and TNF-*α* endothelial cell expression was upregulated and suggested that hypertension activates endothelial cells to increase the expression of adhesion molecules and cytokines and induces neutrophil and monocyte adhesion and migration, resulting in endothelial cell injury and increased permeability of endothelial cells, which results in hypertensive arterial disease [18].

HCH-SBI is an acute onset and rapid progression vascular disease, and it has a serious impact on the quality of life and safety of the patient because it can result in higher level brain function disturbances such as coma, aphasia, dementia, and epilepsy. Explaining the mechanism of action of* Nao-Xue-Shu* oral liquid in TCM theory may be difficult to understand for most Western doctors [9]. HCH-SBI in TCM is explained as “*apoplexia*” and an “*attack on the viscera and bowels*” [19], caused by a* Qi* deficiency, blood stasis, and phlegm. Due to the* Qi* deficiency, the blood stasis and phlegm obstruct the internal structure of blood vessels that then intertwist with each other, and the abnormal blood causes intervessel high blood pressure, and forcing the static blood with phlegm out of the blood vessel may break the vessel, leading to hemorrhage [20, 21]. The* blood stasis* and* phlegm* may be expressed as “inflammatory” and “abnormal blood viscosity” in Western medicine [9], In TCM theory, if* blood stasis* is accompanied by* phlegm*, it can lead to a more significantly damaged lesion in the brain [19]. This is the mechanism that explains why HCH patients often also have advanced neuronal damage, including coma, aphasia, and epilepsy. Physiologically, cleaning and powerful* Qi* (*Qing-yang Qi*) can supply energy to the brain to maintain its function and collect and modulate the blood and force it to circulate in the correct way in brain blood vessels [9, 19, 20]. If the circulation has been obstructed by the blood stasis with phlegm, the occlusion of blood vessel orifices will occur and the power of* Qi* will decrease;* Qing-yang Qi* is also like nutrition for the brain; if it cannot rise, it can lead to the brain lacking sufficient energy to maintain awaking and thinking and can even cause lethargy and coma. If the* Qing-yang Qi* deteriorates further, blood pressure may not be maintained, hypotension will occur, and then SBI may develop. When treating this disease, we should consider three TCM pathogenic matters:* Qi*, blood stasis, and phlegm. First, we should eliminate* Qing-yang Qi*, which can modulate blood circulation and control or decrease bleeding.* Astragalus* root as a major component in* Nao-Xue-Shu* oral liquid can provide a stronger* Qing-yang Qi* [9].* Qi* also provides energy to raise the nutrient level in the blood to the brain when treating the ischemia and improves the level of consciousness [20]. In TCM,* Qi* can improve circulation throughout the entire system and enhances metabolism. The other main component in the oral liquid is* Hirudo*, a type of earthworm that has been used for more than one thousand years in China, which can rapidly eliminate blood stasis and treat the second pathogenic condition, that of blood stasis [21, 22], without any side effects such as bleeding. Other than these two components, the* Nao-Xue-Shu* oral liquid formulation contains 5 other TCM herbs that can help increase* Qi*, remove blood stasis and phlegm, assist the body to excrete the pathogenic metabolites of blood stasis and phlegm, and finally decrease the IL-4, IL-6, IL8, and TNF-*α* levels [23]. Several studies [24–27] have provided strong evidence that TCM, which promote blood circulation to remove blood stasis functions, could diminish inflammation by decreasing IL-6, IL-8, and TNF-*α* factors. In fact,* Nao-Xue-Shu* oral liquid contains two famous prescriptions of TCM. One is* Bu-Yang-Huan-Wu* decoction, which originated in the* Qing Dynasty* (about 185 years ago in 1830) and has been used frequently to treat stroke in China and Asia [27]. The other is* Da-Huang-Shu-Chong* pill, which comes from the very famous TCM text* Jin-Gui-Yao-Lue* (By* Zhang Zhongjing*, about 1700 years ago) and has been used to remove blood stasis from the body [28, 29]. The combination of these 2 prescriptions is the most effective treatment for treating HCH with SBI. Clinical pharmacological studies have confirmed that* Nao-Xue-Shu* oral liquid accelerates the absorption of hematoma in the brain of rats, reduces edema around the hematoma accelerating fibrinolysis and inhibiting thrombosis, increases cerebral blood flow, and improves brain blood and oxygen supply, thereby improving blood circulation and promoting the absorption of hematoma [30].

In this study in patients with HCH, compared with the control group, patients in the treatment group had a markedly lower incidence of SBI.* Nao-Xue-Shu* oral liquid may decrease the mass effects of hematoma, improve the absorbance of hemorrhage, eliminate the toxic stimulation of peripheral brain tissue, and inhibit the accumulation of inflammatory factors. Thus, it can prevent the occurrence of SBI as well as treat HCH-SBI. We did not observe any more side effects based on the results of the laboratory tests.

Chinese medicine has the distinctive function of modulating the body or dealing with diseases, including treating brain problems [31, 32]. We are still unable to show how the ingredients pass through the blood-brain barrier (BBB), but they have been used in many countries for treating many diseases [14].* Nao-Xue-Shu* oral liquid contains a type of worm and this may be one problem in terms of ethics or acceptability as it may be difficult to introduce such a treatment into some foreign countries, although worms are frequently used in TCM treatments and TCM researchers in China have demonstrated that they are harmless and safe. Non-RCT and insufficient cases are the other shortages of our study. In order to validate the causes of the disease based on clinical data, large-scale, multicenter, double-blind randomized control studies may be needed to verify the effectiveness of* Nao-Xue-Shu* oral liquid in the treatment of HCH and HCH-SBI.

## Figures and Tables

**Table 1:** General characteristics of the two groups.

	Treatment	Control	Statistic	*P* value	95% CI (treatment/control)
M/F	47/31	50/30	*χ* ^2^ = 0.084	0.772	—
Age (y)	64.19 ± 7.26	63.08 ± 9.81	*t* = 0.807	0.421	62.58–65.81/60.93–65.23
Onset time BP (SBP) (mmHg)	173.37 ± 29.55	169.89 ± 30.62	*t* = 0.727	0.469	166.81–179.93/163.18–176.60
Onset time BP (DBP) (mmHg)	109.83 ± 12.69	110.31 ± 13.15	*t* = 0.233	0.816	107.01–112.65/107.42–113.19
Volume (mL)	38.64 ± 14.52	36.91 ± 19.37	*t* = 0.634	0.527	35.42–41.86/32.67–41.15
Outside of putamen (putamen and external capsule) (*n*)	37	36	*χ* ^2^ = 0.560	0.967	—
Inside of putamen (globus pallidus and internal capsule) (*n*)	19	21			
Subcortex (*n*)	9	10			
Cerebellum (*n*)	7	5			
Thalamus and broken into ventricles (*n*)	7	8			

**Table 2:** Quantitative changes of interleukin 6 (IL-6), tumor necrosis factor *α* (TNF-*α*), and the volume of hematoma before and after the additional treatment in the treatment and control groups.

Group	Time points	IL-6 (pg/mL)	TNF-*α* (pg/mL)	Volume (mL)
Treatment (*n* = 74)	Baseline	84.26 ± 12.47	186.92 ± 36.55	38.64 ± 14.52
	Week 1	53.83 ± 27.64^*∗*#^	121.34 ± 27.66^*∗*#^	29.37 ± 10.58^*∗∗*#^
	Week 2	21.08 ± 15.39^*∗∗∗*###^	73.59 ± 36.72^*∗∗∗*###^	17.81 ± 9.37^*∗∗∗*##^

Control (*n* = 72)	Baseline	87.09 ± 12.47	183.33 ± 43.35	36.91 ± 19.37
	Week 1	72.63 ± 25.58	169.28 ± 35.91	31.96 ± 13.86
	Week 2	50.71 ± 19.07^*∗*^	108.35 ± 45.49^*∗∗*^	25.95 ± 12.36^*∗∗*^

Note: ^*∗*^ *P* < 0.05, ^*∗∗*^ *P* < 0.01, and ^*∗∗∗*^ *P* < 0.001 compared with baseline of the same group; ^#^ *P* < 0.05, ^##^ *P* < 0.01, and ^###^ *P* < 0.001 compared with control group for the same time points.
